# Organic Iron Supplementation in Cows and Its Impacts on Animal Health and Production

**DOI:** 10.3390/ani15233373

**Published:** 2025-11-21

**Authors:** Natalia Gemelli Corrêa, Maksuel Gatto de Vitt, Guilherme Luiz Deolindo, Gilnei Bruno da Silva, Daiane Manica, Margarete Dulce Bagatini, Miklos Maximiliano Bajay, Aleksandro Schafer da Silva

**Affiliations:** 1 Departamento de Zootecnia, Universidade do Estado de Santa Catarina (UDESC), Chapecó 89815-630, Brazil; natalia.gc703@gmail.com; 2 Programa de Pós-Graduação em Zootecnia, Universidade do Estado de Santa Catarina (UDESC), Chapecó 89815-630, Brazil; 3 Programa de Pós-Graduação Multicêntrico de Bioquímica e Biologia Molecular, Universidade do Estado de Santa Catarina (UDESC), Lages 88520-000, Brazil; 4 Programa de Pós-Graduação em Bioquímica, Universidade Federal de Santa Catarina, Florianópolis 88040-900, Brazil; 5 Programa de Pós-Graduação em Ciências Biomédicas, Universidade Federal Fronteira Sul, Chapecó 89815-899, Brazil; 6 Departamento de Ciências Biológicas, Universidade do Estado de Santa Catarina (UDESC), Laguna 88790-000, Brazil

**Keywords:** micromineral, Jersey, immunity, nutraceutical

## Abstract

This study evaluated whether iron supplementation in the form of chelated minerals in cows in the final third of lactation has a positive effect on iron bioavailability, immunity, oxidative status, milk quality, and biochemical and hematological parameters, as well as production efficiency and fecal microbiota. Twenty-four multiparous Jersey cows, with 210 ± 18 days in milk (DIM), an average production of 25 kg, and 4 ± 0.6 months of gestation, were divided into two groups: Control (*n* = 12, without supplementation) and Iron (*n* = 12, supplemented with 30 mg of iron/kg of dry matter (600 mg/animal/day)). Blood and milk samples were collected on days 1, 16, 29, and 42 of the experiment. Supplemented animals had higher serum iron concentrations and a higher unsaturated iron-binding capacity, especially on day 42. Higher iron content in milk was also observed. A higher granulocyte count was observed in the iron group, as well as a lower number of lymphocytes compared to the control, which may indicate immunosuppression associated with iron supplementation. Fructosamine levels were significantly lower in the iron group animals on days 14 and 28, suggesting a possible alteration in glucose metabolism. In contrast, levels of the liver enzymes AST and ALT increased significantly in the group supplemented with iron on days 28 and 42, indicating potential liver overload or injury. Iron supplementation significantly increased levels of reactive oxygen species and thiobarbituric acid-reactive substances, as well as superoxide dismutase activity in the blood. Iron supplementation altered gut microbial diversity, promoting dysbiosis characterized by increased alpha diversity and enrichment of transient colonization by *Nitratireductor*, *Brevundimonas*, *Flavobacterium* and *Sporosarcina*. Lower milk production was observed in the iron-supplemented cows in the last 10 days of the experiment, which is related to the occurrence of disease in nine cows in this group: seven with mastitis and two with intestinal peristalsis paralysis. Based on these results, we conclude that chelated iron supplementation at a dose of 600 mg/animal/day should not be used because it harms cow health and productivity.

## Simple Summary

The use of iron as a dietary supplement for dairy cows is a new research topic, with few studies. Chelated iron supplementation in cows in the final third of lactation increased serum and milk levels of the mineral, demonstrating its high bioavailability and nutraceutical potential for milk enrichment. However, the strategy also caused adverse effects, such as immunological alterations consistent with the activation of the innate response and possible adaptive immunosuppression, increased markers of oxidative stress, elevated liver enzymes, reduced milk production, and a higher incidence of clinical disorders. Supplemental iron intake modulated the gut microbiota, increasing opportunistic and potentially pathogenic microorganisms.

## 1. Introduction

The high metabolic demands of high-producing dairy cows place significant pressure on fundamental physiological systems, such as hematopoiesis and the immune response. Increased plasma volume and milk production rate can dilute erythrocyte and hemoglobin concentrations, predisposing these cows to subclinical anemia [1]. Furthermore, the immunological challenge faced by high-performance cows can compromise the effectiveness of innate and adaptive immunity, increasing the risk of persistent infections and inflammation [2]. These factors, combined with the low intake of certain micronutrients, reinforce the need for nutritional strategies that prioritize not only productivity but also physiological balance.

In this context, chelated minerals have stood out for their greater bioavailability and functional efficacy compared to traditional inorganic forms [3]. Minerals, when bound to amino acids, peptides, or other organic ligands, form more stable complexes in the gastrointestinal tract, making them less susceptible to antagonistic interactions with other nutrients and favoring their absorption. Furthermore, they have a positive influence on immunity and oxidative metabolism [4]. In the specific case of iron, this mineral plays an essential role in erythropoiesis, mitochondrial function, cellular immunity, and antioxidant activity. On the other hand, excess iron can trigger pro-oxidant effects, promote bacterial growth, and aggravate systemic inflammatory conditions [5,6]. Therefore, supplementation with organic iron may be a promising alternative for correcting subclinical deficiencies and preventing anemia in high-producing cows, in addition to potentially increasing iron levels in milk, adding nutraceutical value to the feed and benefiting vulnerable populations, such as children [6]. However, organic iron can have high bioavailability, which can create pro-oxidant potential, and its action on immune and inflammatory pathways can have negative impacts on animal health.

Supplementation of 600 mg of iron per day as an amino acid–iron complex in lactating dairy cows tended to increase milk production [7]. Another study found no effect of iron supplementation on milk production, milk composition, or dry matter intake; however, it reduced milk somatic cell count [8]. In young ruminants supplemented with iron, increases in red blood cell levels, hemoglobin, hematocrit, mean corpuscular hemoglobin, and mean corpuscular concentration were distributed [9,10,11]. Therefore, our hypothesis is that iron supplementation could improve the blood parameters of the cows used in this study, thereby increasing milk production by promoting oxygenation of the animal’s body. Therefore, the objective of this study was to evaluate the impacts of chelated iron supplementation on the mineral’s bioavailability, hematological, immunological, biochemical, and oxidative parameters, and milk production in dairy cows, as well as feces microbiota.

## 2. Materials and Methods

This study was conducted at the Experimental Farm of the Western Higher Education Center of the Santa Catarina State University in Guatambú, Santa Catarina, Brazil. The experimental protocol (no. 6332260824) was approved by the Animal Use Ethics Committee of the Santa Catarina State University, Brazil.

### 2.1. Mineral

The organic iron used in this study belongs to the Elements line from YesSinergy (São Paulo, SP, Brazil), a company specializing in biotechnology applied to animal nutrition and health. The product, called Elements Iron, is composed of chelated iron amino acids, a highly bioavailable form of the mineral.

### 2.2. Animals and Facility

Twenty-four multiparous Jersey cows in the final third of lactation were used in this study. They were 210 ± 18 days in milk (DIM), had an average production of 25 kg, and were 4 ± 0.6 months pregnant. The animals, housed in a compost barn, were milked using a DeLaval VMS™ V300 robotic milker (DeLaval do Brazil, Jaguariúna, São Paulo, Brazil), in a free-range milking system. The facility featured manually activated fans to control the thermal environment.

### 2.3. Experimental Design and Diet

The study lasted 42 days, with the first 16 days considered an adaptation period. The experimental design was completely randomized with varying numbers of replicates. The cows were divided into two groups based on milk production over the previous seven days, the number of days in lactation (DIM) for each cow, and individual hematocrit analysis, ensuring homogeneity and minimizing interindividual variation in these factors. The animals were divided into two groups: the iron group (*n* = 12) which received iron supplementation in the form of chelated iron amino acids (30 mg iron/kg dry matter, equivalent to 600 mg/animal/day), and the control group (*n* = 12), which received no iron supplementation. The supplementation dose was established based on the study by DeFrain et al. [7], who used the same iron level in dairy cows. Five days after the start of the experiment (adaptation period), one of the cows in the treatment group contracted babesiosis followed by anaplasmosis, becoming debilitated due to lack of feed, weight loss, and a drastic reduction in milk production; therefore, data from this animal were not included in this research. Thus, for the treatment group, we used data from only 11 cows.

The basal diet was formulated based on National Academies of Sciences, Engineering, and Medicine (NASEM) recommendations to meet the nutritional requirements of multiparous lactating Jersey cows with an average milk production of 25 kg per day, a fat content of 4.50%, a true protein of 3.30%, and approximately 210 days in lactation. A non-commercial vitamin-mineral core from CooperAlfa (Chapecó, Santa Catarina, Brazil) was used in the formulation, without the inclusion of inorganic iron. The diet used in the experiment, as well as its calculated composition, is presented in Supplementary Materials (Table S1). The average nutritional requirement of adult dairy cows is approximately 22 mg iron/kg of dry matter according to NASEM while the basal diet of the control group contained 27.21 mg iron/kg DM (Table S1). Therefore, the dose of 600 mg/cow/day is a supplement, not a correction of iron deficiency.

The animals were housed in a single collective pen, but feed delivery was individually managed. A concentrate referred to as basal concentrate was formulated and provided to the control group. The same concentrate was used for the treatment group, but with the inclusion of chelated iron in its formulation. Morning (7:40 a.m.) and afternoon (3:00 p.m.) meals were offered in individual feeding troughs, for approximately 1.5 h and consisted of silage, hay, and the respective concentrate according to each experimental group. At night, feeding took place in smart feeders (Intergado^®^, Ponta, Betim, Minas Gerais, Brazil), with free access only to authorized animals (during night feeding, the diet consumed was not supplemented with iron; that is, all animals consumed the same diet). Additionally, during milking, the animals received up to 2.0 kg/day of a pelleted concentrate specifically for robotic milking, Nutrialfa^®^ Bovino Robô AP (CooperAlfa, Bom Jesus, Santa Catarina, Brazil). Iron supplementation was administered to the experimental group, in which the organic mineral was incorporated into the concentrate during its production. The animals in the control group received exclusively the basal diet, while those in the iron-supplemented group received the same diet with the addition of the mineral in chelated form. The supplementation level followed the recommendations of DeFrain et al. [7], who used iron complexed with amino acids (IRN, Availa-Fe, Zinpro Corp., Eden Prairie, MN, USA) in a study conducted with 506 multiparous lactating Holstein cows.

To assess iron availability in the concentrates, analyses were performed on the basal concentrate, as well as on the same concentrate after the addition of the chelated organic mineral. Considering that ingredients such as corn, soybean meal, and other dietary components already naturally contain traces of inorganic iron, these analyses were essential for determining the effective mineral composition of the concentrate fed to the animals. The iron levels detected in the concentrates are presented in Table 1.

### 2.4. Data and Sample Collection

Daily milk production, pelleted concentrate intake, and body condition score (BCS) for each cow were automatically monitored using a robotic milking system. BCS was determined daily by capturing three-dimensional images of the cows’ lumbosacral region using a camera (DeLaval BCS™, Delaval do Brazil, Jaguariúna, São Paulo, Brazil) positioned at the exit of the milking robot. The images obtained allowed the system to classify the animals on a BCS scale from 1 to 5, with 1 indicating animals in poor body condition (thin) and 5 indicating animals in high body condition (fat).

Individual feed consumption was estimated based on the difference between the supply and the leftovers in individual feeders, when available. Leftovers were weighed daily before offering the second feed of the day. Because the animals consistently occupied the same feeders, accurate feed consumption monitoring was possible. It should be noted that the 600 mg/animal/day supplementation was divided into two feedings, administered in the feeding lane, in individual feeders, to ensure the planned iron intake. During the nighttime feeding in the automatic feeders, the diet was the same for cows in both groups, without iron supplementation.

During the experimental period, blood, milk, and feed samples were collected on days 1, 16, 29, and 42. Blood collection was performed by venipuncture of the coccygeal vein using a cannon and needles attached to vacuum tubes (Vacuplast^®^, Cral, Cotia, São Paulo, Brazil). For hematological analysis, 4 mL of blood was collected in tubes containing anticoagulant (EDTA K3), and for biochemical analysis, 10 mL was collected in tubes with clot activator to later obtain serum. After collection, the samples were kept refrigerated (<5 °C) and transported to the laboratory. Hematological analysis was performed immediately upon arrival at the laboratory. To separate the serum, the tubes were centrifuged at 7000 rpm for 10 min at room temperature using a QUIMIS^®^ centrifuge (model Q222T, Diadema, São Paulo, Brazil). The serum obtained was then transferred to microtubes and stored at −20 °C until laboratory analysis.

Milk collections were performed individually for each animal using specific tubes attached to the automatic collector of the milking robot. Samples for milk quality analysis received a preservative tablet (Brononata^®^, Laborclin, Pinhais, Brazil) and were stored at 4 °C until shipment to the responsible laboratory. To evaluate the possible nutraceutical effect of iron supplementation, milk samples were transferred to microtubes and stored at −20 °C until iron quantification was performed. On the 42nd experimental day, fecal samples were collected using sterile swabs (3M™ Quick Swabs, Saint Paul, MN, USA) for qualitative and quantitative characterization of the intestinal microbiota. Analyses were performed using metagenomics, using 16S rRNA gene sequencing, conducted by the BPI—Biotechnology Research and Innovation^®^ laboratory, located in Botucatu, São Paulo, Brazil.

Throughout the experimental period, feed samples were collected, both as individual ingredients and as partially mixed rations. The samples were initially stored at −20 °C and were subsequently thawed and homogenized to form a representative sample of the period, based on collections made at different time points.

### 2.5. Feed Composition Analysis

To determine the chemical composition of the supplied feeds, the samples were initially weighed before and after pre-drying in an oven with forced air circulation at 55 °C for 72 h to estimate the partial dry matter content. After partial drying, the samples were ground in a Wiley mill (Marconi^®^, model MA340, Marconi, Piracicaba, Brazil) using a 1 mm mesh sieve to standardize the particle size for subsequent laboratory analysis.

The ground samples were then oven-dried at 105 °C until a constant weight was obtained to determine the total dry matter content. Mineral matter was determined by incinerating the samples in a muffle furnace at 600 °C until the organic matter was completely burned. Crude protein quantification was performed using the micro-Kjeldahl method, according to the official methodology described by AOAC 2001.11 [12]. The ether extract content was obtained using an automatic fat extractor (VELP Scientifica^®^, model SER 158, Usmate, Italy), following the official AOAC 2003.05 method [12], replacing diethyl ether with petroleum ether. Neutral detergent fiber (NDF) and acid detergent fiber (ADF) analyses were conducted using a fiber analyzer (ANKOM^®^ 200, Ankom, New York, NY, USA) according to the equipment-specific protocols, with the following adaptation: replacing the ANKOM^®^ filter bags with bags made of nonwoven fabric. The methodologies were based on the method described by researchers [13] for NDF, and the official AOAC method 973.18 [12] for ADF.

To assess iron availability in concentrates, analyses were performed on the basal concentrate, as well as on the same concentrate after the addition of the chelated organic mineral. Considering that ingredients such as corn, soybean meal, and other dietary components already naturally contain traces of inorganic iron, these analyses were essential for determining the effective mineral composition of the concentrate fed to the animals. In addition to iron in the concentrate, aluminum, boron, copper, manganese, and zinc were measured using the methodology described in the Brazilian Compendium of Animal Feed [14].

The chemical composition of the main ingredients fed, including silage, hay, concentrate, and pelleted concentrate fed via milking robot, are detailed in Table 1, as are the iron levels detected in the concentrates.

### 2.6. Complete Blood Count

The complete blood count was performed using an automated hematology analyzer (EQUIP VET^®^ 3000, Equip, São Paulo, Brazil). Hematological analyses included total leukocyte count (×10^3^/µL), including differentiation into lymphocytes, granulocytes, and monocytes (×10^3^/µL). Furthermore, erythrocytes (×10^6^/µL), platelets (×10^3^/µL), hemoglobin concentration (g/dL), and hematocrit (%) were quantified. These variables were used to assess the hematological profile of the animals throughout the experiment.

### 2.7. Serum Biochemistry

Serum biochemistry analysis was performed using an automated analyzer (Zybio^®^ EXC 200, Equip, Itatiba, São Paulo, Brazil), with the aid of specific commercial kits (Analisa^®^, Belo Horizonte, Brazil). The measured variables included creatine kinase (CK) and cholinesterase enzymatic activity (U/L), as well as serum concentrations of albumin (g/dL), cholesterol (mg/dL), ferritin (µg/L), iron (µg/dL), fructosamine (µmol/L), gamma-glutamyltransferase (GGT; U/L), C-reactive protein (CRP; mg/L), unsaturated iron-binding capacity (UIBC; µg/dL), total protein (g/dL), aspartate aminotransferase (AST; U/L), alanine aminotransferase (ALT; U/L), and urea nitrogen (mg/dL). Globulin levels were obtained by indirect calculation, based on the difference between total protein and albumin concentrations.

### 2.8. Oxidative Status

The oxidative status variables assessed in blood serum were reactive oxygen species (ROS), thiobarbituric acid-reactive substances (TBARS), myeloperoxidase (MPO) activity, total thiols (PSH), and superoxide dismutase (SOD). Analyses were performed in triplicate using specific biochemical methodologies.

The formation of reactive oxygen species (ROS) was estimated using the fluorimetric protocol established by researchers [15], in which 10 µL of serum was incubated with an equal volume of 2′,7′-dichlorofluorescein diacetate (DCFH-DA, 7 µM) and 240 µL of phosphate-buffered saline (PBS). After 30 min of incubation at 37 °C, the final product of DCFH-DA oxidation, dichlorofluorescein (DCF), was measured by fluorescence with excitation at 488 nm and emission at 525 nm (Thermo Scientific™ Varioskan™ LUX, Waltham, MA, USA). The results were expressed as a percentage of the fluorescence intensity relative to the analytical control.

The concentration of thiobarbituric acid-reactive substances (TBARS), whose main marker is malondialdehyde (MDA), was used to measure lipid peroxidation, which reflects the oxidative degradation of polyunsaturated fatty acids, as described in [16]. For this purpose, 20 µL of serum was homogenized with 55 µL of distilled water, 100 µL of orthophosphoric acid (0.2 M), and 25 µL of thiobarbituric acid (TBA, 0.1 M). After 45 min of incubation at 37 °C, the reading was performed at 532 nm, with the results expressed in nM MDA/mL.

Myeloperoxidase (MPO) enzyme activity was analyzed using a modified peroxidase system, according to [17]. In the presence of H_2_O_2_ as an oxidizing agent, MPO catalyzes the oxidation of phenol and 4-aminoantipyrine (AAP), resulting in the formation of quinoneimine, a colored product with a maximum absorbance at 492 nm. For the reaction, 12 µL of serum was mixed with 148 µL of AAP in phenol solution (2.5 mM AAP and 20 mM phenol) and 17 µL of H_2_O_2_ solution (17 mM). After incubation for 30 min at 37 °C, the samples were read in a spectrophotometer, and the results were expressed as µM quinoneimine per mg of protein produced in 30 min (μMq/mg/30 min).

Protein thiol content was determined based on the method of Ellman [18], with modifications. For PSH analysis, 30 µL of supernatant was added to 200 µL of potassium phosphate buffer (PPB, 1 M, pH 6.8) and 20 µL of 5,5′-dithiobis(2-nitrobenzoic acid) (DTNB), with immediate reading at 412 nm. The result was expressed in µM/L, using a cysteine standard curve as a reference.

SOD activity was determined based on the inhibition of the superoxide radical reaction with adrenaline, as described by researchers [19]. In this method, the SOD present in the sample competes with the detection system for the superoxide radical. The results were expressed in units of SOD enzyme activity per milligram of protein (U/mg of protein).

### 2.9. Iron Levels in Serum and Milk

Serum was obtained through enzymatic coagulation of milk. For each animal, 2 mL of milk was collected and placed in properly labeled Eppendorf microtubes. Then, 1.4 µL of commercial rennet Ha-La (Chr. Hansen Indústria e Comércio Ltd.a, Valinhos, São Paulo, Brazil), a liquid chymosin-based coagulant, was added according to the manufacturer’s recommendations. After addition of the coagulant, the samples were incubated in a water bath at 37 °C for 20 min to promote coagulation. Subsequently, the samples were centrifuged at 4500 rpm for 20 min to separate the serum, as described by Sant’Ana and Birgel [20].

### 2.10. Milk Quality

Milk quality analyses were conducted by the Centralized Milk Analysis Laboratory, part of the Paraná Dairy Herd Analysis Program, which is accredited by the Ministério da Agricultura, Pecuária e Abastecimento (MAPA, Brasilia, Brazil). Total protein, lactose, fat, total solids, and milk urea nitrogen (MUN) contents, expressed as percentages, were determined using Fourier transform mid-infrared spectrometry, in accordance with ISO 9622/IDF 141:2013 (ISO, 2013). Somatic cell count (SCC), expressed in ×10^3^/mL, was performed by flow cytometry, according to the procedures described in ISO 13366-2/IDF 148-2:2006 (ISO, 2006).

### 2.11. Productive Performance

Dry matter intake (DMI) was calculated by adding the partial diet intake from individual feeders, the intake from Intergado^®^ feeders (Força, Betim, MG, Brazil), and the pelleted concentrate supplied by the milking robot. All values were converted to a dry matter basis using the results of the chemical analyses of the feed. Feed efficiency (FE) was determined by the relationship between milk production and DMI, according to the formula: FE = Milk production (kg/day)/DMI (kg/day).

### 2.12. Fecal Microbiota

On day 42, feces were collected and stored in 3M™ Quick Swabs for qualitative and quantitative detection of microorganisms using metagenomics by sequencing the 16S rRNA gene, performed by the laboratory BPI—Biotechnology Research and Innovation^®^, Botucatu, São Paulo, Brazil. Total DNA was extracted from 200 mg (wet weight) of samples with the ZR Fungal/Bacterial DNA MiniPrep kit (Zymo Research, Irvine, CA, USA) and primers 341F (5′-CCTAYGGGRBGCASCAG-3′) and 806R (5′-GGACTACNNGGGTATCTAAT-3′) were selected to amplify the V3–V4 region of bacterial 16S rRNA gene by the polymerase chain reaction [21]. Libraries were quantified by qualitative polymerase chain reaction using the Kapa Library Quantification Kit (Illumina, San Diego, CA, US) following the manufacturer’s recommendations. Samples were normalized to a final concentration of 2 nM and sequenced with an Illumina MiSeq for 250 cycles from each end.

Raw sequencing data were analyzed using Mothur v.1.48.3 [22], following the established MiSeq Standard Operating Procedure [23]. Taxonomic classification of representative oligotype sequences was performed against the SILVA reference database (release 132) [24] and Greengenes2 database [25]. The resulting clustered operational taxonomic unit (OTU) data was subsequently imported into R version 4.5.1 (R Core Team, 2025) for downstream processing with the Phyloseq package (v1.52) [26]. Microeco package (v1.15.0) was applied to aggregate taxonomic abundance data at the genus and species level, and relative abundance profiles of the ten most prevalent genera and species were generated by averaging across animals and experimental groups [27]. ggplot2 package (v4.0.0) was used to build graphical plots [28].

Alpha diversity within bacterial communities was assessed using violin plots of the inverse Simpson diversity index. Differences in microbial community composition among samples were examined using Principal Coordinate Analysis (PCoA) constructed from Bray–Curtis dissimilarity matrices. This ordination approach enables visualization of beta diversity patterns, highlighting compositional dissimilarities between groups based on overall taxonomic profiles.

To identify bacterial taxa that differed significantly in relative abundance among experimental groups, differential taxonomic analysis was performed using the LEfSe (Linear Discriminant Analysis Effect Size) algorithm [29]. This approach combines non-parametric statistical testing with linear discriminant analysis to detect features that best explain group-level differences, thereby revealing discriminative microbial taxa and patterns of community structure across treatments.

### 2.13. Statistical Analysis

A descriptive analysis of the data was performed, followed by the normality test (Shapiro–Wilk) and measurement of residual error. Data that were not normally distributed (total leukocytes, monocytes, and iron in milk) were log-transformed and normalized for subsequent analyses using the SAS ‘MIXED’ procedure (SAS Inst. Inc., Cary, NC, USA; version 9.4). The variables mean milk production, lactation persistence, and feed efficiency were tested for a fixed treatment effect, using animal (treatment) as a random effect. The remaining data were analyzed as repeated measures and tested for fixed effects of treatment and day, as well as the treatment × day interaction, where cow (treatment) was the random effect. The results of d1 were included as an independent covariate; d1 was removed from the data set to generate the mean by treatment but retained as a covariate. Furthermore, the covariance structure was first-order autoregressive, selected according to the lowest Akaike information criterion. Means were separated using the PDIFF method (Student’s *t*-test), and all results were reported as LSMEANS followed by the standard error of the mean (SEM). Significance was defined as *p* ≤ 0.05, and a trend when *p* > 0.05 and ≤0.10.

## 3. Results

### 3.1. Clinical History of the Experiment

The cows in the control group remained apparently healthy during the experimental period. The iron-supplemented cows, however, experienced mastitis (7/11 animals) and interrupted intestinal peristalsis (2/11 cows). All cases of mastitis were clinical, with diagnoses occurring on day 18 (animal 1), day 28 (animals 2 and 3), day 32 (animal 4), day 33 (animal 5), and day 36 (animals 6 and 7). Intestinal paralysis occurred in one of the cows 30 days into the experiment. It was detected early in the morning (the previous day, with no changes in production, consumption, or behavior). It progressed rapidly, leaving the animal with motor incoordination, difficulty standing, and fever. The animal then remained in lateral recumbency (less than 2 h after the motor incoordination was identified). It remained in this position for 36 h, until it began to respond to medication (antibiotic therapy (penicillin for 7 days), fluid therapy with electrolyte replacement, and analgesic (dipyrone) and resumed eating. The animal needed to be suspended to stand and walk again; it remained active (experimental feeding was suspended and the animal was removed from the experiment from the day it became ill). On day 35 of the experiment, a second animal developed intestinal paralysis, when clinical signs were identified in the afternoon (the cow had normal behavior and feeding behavior during the morning). It was observed that the animal stopped eating, began to exhibit muscle tremors, and began to exhibit motor incoordination. The same protocol was initiated for the other cow, which allowed the cow to remain upright and alert. The cow resumed feeding the following day, but in quantities less than 50% of what was consumed before the animal became ill. The cows with mastitis received two commercial intramammary infusions at 24 h intervals (Mastijest forte^®^: combination of tetracycline, neomycin, bacitracin and prednisolone) combined with an anti-inflammatory (Maxicam^®^: meloxicam), and the milk was discarded for 9 days; all animals responded to the treatment. Only two cows in the iron-supplemented group showed no clinical manifestations. The experiment was scheduled to last 56 days, but due to the events reported here, we decided to discontinue the study. After the study was completed, the animals were monitored, without any apparent clinical changes.

### 3.2. Blood Count and Serum Biochemistry

According to the blood count results presented in Table 2, no significant differences were observed in total leukocyte counts between the organic iron-supplemented and control groups (*p* > 0.05). However, lymphocytes showed a treatment × day interaction (*p* = 0.05), although the isolated effect of treatment was not significant (*p* = 0.12). On day 42, the Iron group had a significantly lower number of lymphocytes compared to the control group, demonstrating a possible modulation in the leukocyte profile. Furthermore, a significant difference was observed in the mean granulocyte count, with an effect for both treatment (*p* = 0.05) and the treatment × day interaction (*p* = 0.01), with the animals supplemented with organic iron having a higher number of cells compared to the control group. The difference was most evident on day 42, when the supplemented group had considerably higher granulocyte values than those observed in the control group.

**Figure 1:**
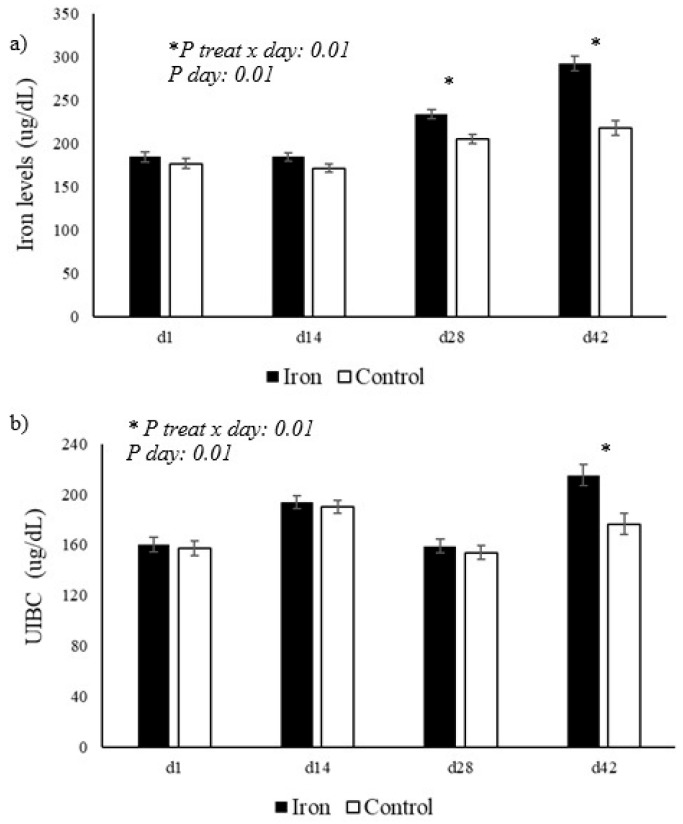
Variation in (**a**) serum iron levels and (**b**) unsaturated iron-binding capacity (UIBC) in Jersey cows supplemented with organic iron. The presence of an asterisk (*) indicates the difference between the groups.

**Figure 2:**
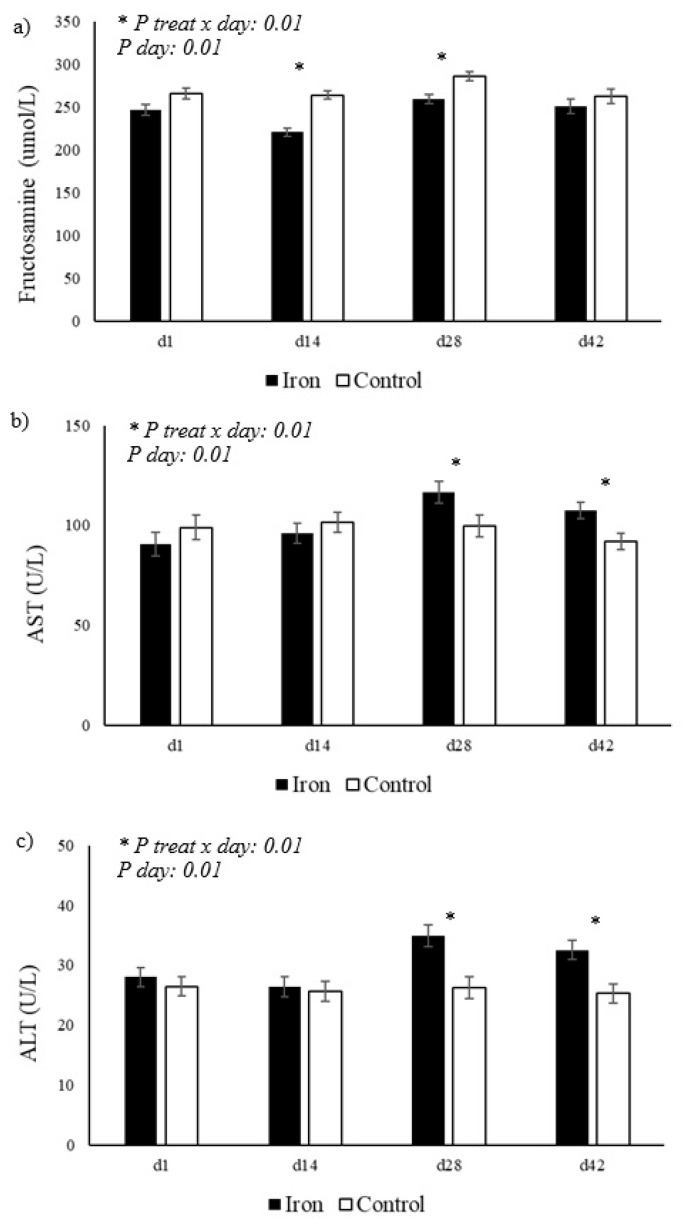
Variation in (**a**) serum fructosamine, (**b**) aspartate aminotransferase, and (**c**) alanine aminotransferase levels in Jersey cows supplemented with organic iron. The presence of an asterisk (*) indicates the difference between the groups.

As shown in Table 2 and illustrated in Figure 1, serum iron levels showed a significant difference between the groups, with higher values in the group supplemented with organic iron (*p* < 0.05). Furthermore, a significant interaction between treatment × day (*p* = 0.01) was observed, indicating that the variation in iron levels over time was distinct between the groups, with a greater increase in the supplemented group on days 29 and 42. For unsaturated iron-binding capacity (UIBC), a significant interaction was also observed for treatment × day (*p* = 0.01), with higher values in the supplemented group on day 42, as shown in Figure 1b. Other biochemical parameters, such as albumin, creatinine, cholesterol, cholinesterase, GGT, total protein, urea, globulin, C-reactive protein, and creatine kinase, did not show significant differences between the groups (*p* > 0.05; Table 2). Additionally, based on the results in Table 2 and illustrated in Figure 2, a significant interaction for treatment × day was observed in the supplemented group for fructosamine levels, as well as aspartate aminotransferase (AST) and alanine aminotransferase (ALT) activity (*p* = 0.01). As shown in Figure 2a, fructosamine levels were lower in the iron-supplemented group on days 16 and 29 compared to the control group. Figure 2b shows that AST activity remained more stable in the control group, while the iron group showed a significant increase on days 29 and 42. Furthermore, Figure 2c shows that ALT activity was significantly higher in the iron group on days 29 and 42 compared to the control group.

### 3.3. Oxidative Status

Oxidative status results are presented in Table 3. For ROS formation, a significant effect of both treatment (*p* = 0.05) and the treatment × day interaction (*p* = 0.023) was observed. ROS levels were higher in the iron group compared to the control group, with statistically significant differences on days 29 and 42, with the supplemented group showing higher values compared to the control group. TBARS levels were significantly different for both the treatment effect (*p* = 0.03) and the treatment × day interaction (*p* = 0.001). The iron group showed higher levels starting on day 16, peaking on day 29 and remaining elevated on day 42. TBARS concentrations were more than double in the iron group compared to the control group, indicating a significant increase in lipid peroxidation associated with organic iron supplementation. Myeloperoxidase (MPO) activities and protein thiol (PSH) levels did not show significant differences. Superoxide dismutase (SOD) activity had a significant effect for the treatment × day interaction (*p* = 0.05). In general, the iron-supplemented group showed higher mean SOD values compared to the control group (Table 3).

### 3.4. Productive Performance and Milk Quality

**Figure 3:**
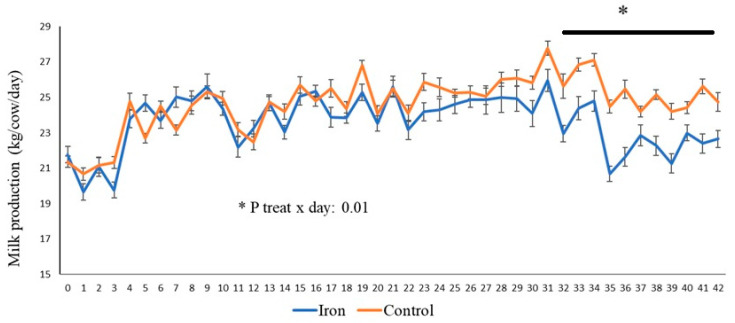
Milk yield in Jersey cows supplemented with organic iron. The presence of an asterisk (*) indicates the difference between the groups.

The results regarding productive performance and feed efficiency are presented in Table 4. No significant differences were observed between treatments for milk production, milk corrected to 4% fat, dry matter intake, or feed efficiency (kg milk/kg DM intake). However, there was a treatment x day interaction (*p* = 0.01) for milk production, indicating that the response to organic iron varied throughout the experiment, as illustrated in Figure 3; i.e., in the last 10 days of the experiment, cows in the supplemented group produced less milk daily. The milk composition of Jersey cows supplemented with organic iron was not significantly influenced by the treatment (Table 4), as the milk fat, protein, lactose, total solids, and urea contents did not show statistical differences between the supplemented and control groups. Somatic cell count (SCC) was higher in supplemented cows at the end of the experiment.

There was a significant effect of treatment on iron levels in milk (*p* = 0.05), with higher concentrations observed in cows supplemented with organic iron, especially on day 42 of the experiment. These results indicate that supplementation was effective in increasing mineral levels in the milk of Jersey cows in the final third of lactation (Table 4).

### 3.5. Gut Microbiota

**Figure 4:**
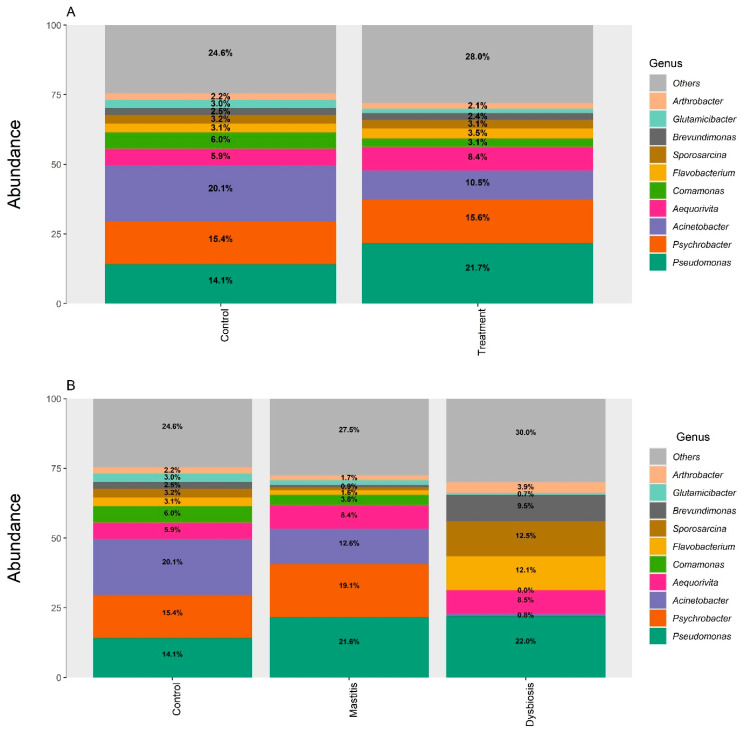
Relative abundances of the 10 most predominant microbial genera in the feces of cows supplemented with organic iron (treatment) compared with the control group (**A**), using the SILVA database. In the treatment group, some cows presented mastitis and dysbiosis; the intestinal microbiota is shown considering these clinical alterations (**B**).

**Figure 5:**
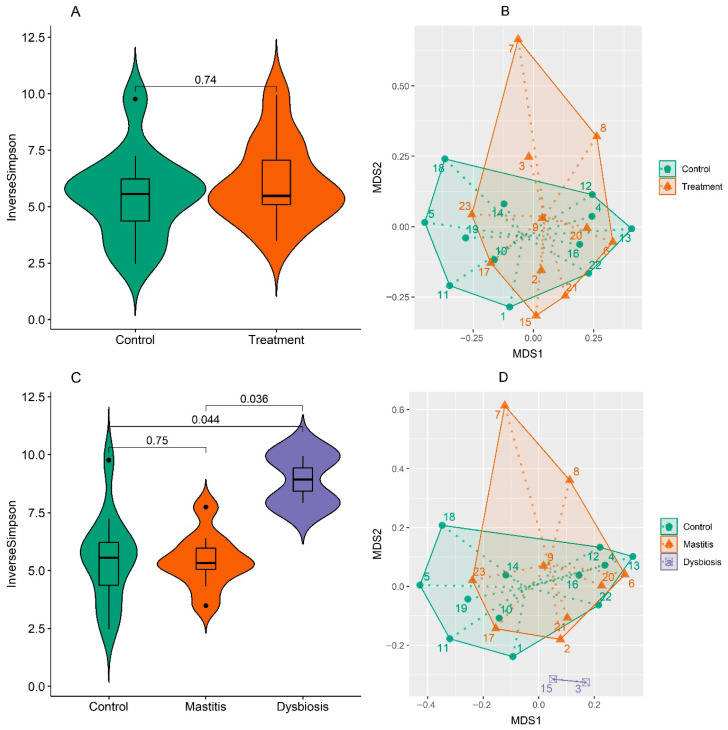
(**A**) Alpha diversity (InverseSimpson) and (**B**) beta diversity (NMDS) did not differ statistically between the control and treatment groups (upper image), using the SILVA database. When the treatment group was analyzed according to clinical presentation, statistical difference was observed for (**C**) alpha diversity (Inverse Simpson) and (**D**) beta diversity (MDS2) when comparing both control vs. dysbiosis and mastitis vs. dysbiosis.

Using the SILVA database, the top 10 most abundant genera of microorganisms in the feces of cows supplemented with organic iron (treatment) were *Pseudomonas*, *Psychrobacter*, *Acinetobacter*, *Aequorivita*, *Comamonas*, *Flavobacterium*, *Sporosarcina*, *Brevundimonas*, *Gluamicibacter*, and *Arthrobacter* (Figure 4). When comparing control and treatment, the proportions of *Pseudomonas* and *Aequorivita* were 53.9% and 42.3% higher in the supplemented cows, respectively. Meanwhile, the genera *Acinetobacter* and *Comamonas* were 52.2% and 51.6% lower in the iron-supplemented cows, respectively. When we analyzed the cows, separating those in the treatment group according to clinical manifestations (mastitis or dysbiosis), we found that the cows that had dysbiosis had significantly altered microbiota, with the *Psychrobacter* genus not being identified; at the same time, the *Flavobacterium*, *Sporosarcina*, and *Brevundimonas* genera were considerably increased in relative abundance compared to their levels in the other animals (Figure 4). Figure S1 shows relative abundance, based on the Green Genes database, for the 20 main species of microorganisms in feces of cows supplemented with iron compared to the most prevalent species in the control, where we highlight those with the greatest difference in proportion: *Acinetobacter idrijaensis* and *Comamonas kerstersii*, which had 51.6% and 53.8% lower abundance in cows supplemented with iron compared to the control. The results of alpha diversity (Shannon) and beta diversity (PCo2) are presented in Figure 5. We found no statistically significant difference between the control and treatment groups using the SILVA database. However, when the treatment group was analyzed considering clinical presentation, beta diversity analysis revealed significant differences between the control and mastitis groups (*p* = 0.049) and between the mastitis and dysbiosis groups (*p* = 0.045). Furthermore, there was a significant difference in alpha diversity when comparing control versus dysbiosis and mastitis versus dysbiosis. In both comparisons, the highest alpha diversity was found in cows with dysbiosis (Figure 5).

**Figure 6:**
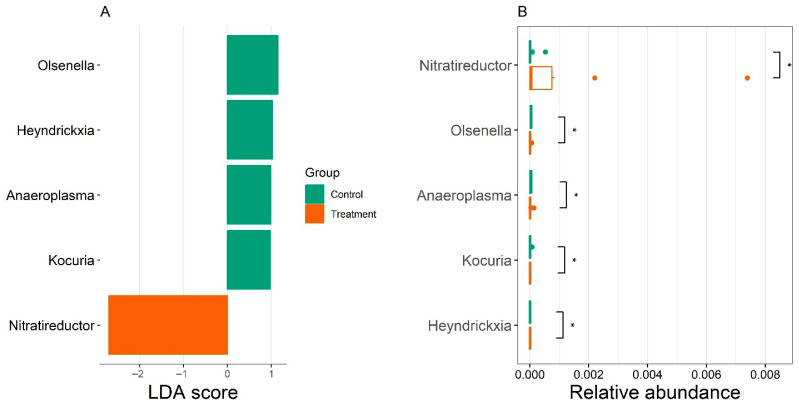
LDA score showing the effect of treatment on five microbial genera using the SILVA database (**A**). A higher relative abundance of the genera *Olsenella*, *Heyndrickxia*, *Anaeroplasma*, and *Kocuria* was observed in the control group, whereas the genus *Nitratireductor* was more abundant in cows from the treatment group (**B**). The presence of an asterisk (*) indicates the difference between the groups.

The LDA score shows the effect of treatment on five genera of microorganisms using the SILVA database (Figure 6). The highest relative abundance of the genera Olsenella, Heyndrickxia, Anaeroplasma, and Kocuria was observed in the control group, with the highest abundance of the genus Nitratireductor in the treatment group.

## 4. Discussion

Although organic iron supplementation increased levels of the mineral in milk, this study showed that this practice can have significant adverse effects on cow health. Iron is an essential nutrient not only for the host but also for several pathogens, which use it as a cofactor for growth and proliferation [30]. The increased availability of iron in the animal’s body can therefore favor the growth of opportunistic bacteria, especially those associated with gastrointestinal tract infections, with the potential to unbalance the intestinal microbiota and increase the colonization of pathogenic microorganisms [31]. At the same time, it contributes to the induction of inflammatory and immunosuppressive processes [32]. This process allows pathogens to sequester the available iron in the body to sustain their multiplication, while the host, as a defense mechanism, reduces the amount of circulating iron. This occurs primarily through the induction of hepcidin, a peptide hormone produced by the liver that blocks the release of iron by cells, hindering microorganisms’ access to the mineral [5]. Thus, excess iron acts as an essential cofactor for the growth of pathogenic microorganisms, favoring infections and contributing to disorders such as mastitis, since inflammation dysregulates the immune system and makes animals more susceptible to opportunistic infections [32]. A study by researchers [6] demonstrated that excess iron in inflammatory situations can induce iron-mediated cell apoptosis, aggravating tissue damage. The study revealed that, in murine models of inflammation and gestational obesity, only the combination of iron overload, and inflammation resulted in significant embryonic toxicity, indicating that the interaction between elevated iron and inflammation potentiates oxidative stress and cell death. This information explains why cows in the iron group experienced mastitis and gastrointestinal tract paralysis.

Accordingly, supplementation with chelated iron resulted in a significant increase in serum iron levels in treated animals, indicating efficient absorption of the mineral. This finding is consistent with the results of researchers [33], who observed elevated serum iron levels in calves fed whole milk and supplemented with a protein–iron complex. This study found that the addition of a protein–iron complex (PIC) significantly impacted indicators of iron metabolism, resulting in an increase in the calves’ whole blood iron concentration. Regarding UIBC, the significant effects of treatment and the treatment × day interaction suggest a dynamic adaptation of iron metabolism over time. The increase in UIBC may reflect a physiological response to increased iron availability, with the body adjusting transferrin production to optimize transport and avoid iron overload. This mechanism is supported by [11], in which the researchers, when supplementing Dalagh lambs with organic iron, observed changes in transferrin levels and transferrin saturation, indicating a modulation of the iron transport system in response to supplementation. Furthermore, studies such as that by Kupczyński et al. [34] demonstrated that supplementation with a protein–iron complex in pre-weaned calves resulted in changes in iron metabolism parameters, including UIBC, suggesting that the form and dosage of supplemented iron can significantly influence these indicators. Importantly, there was an increase in iron in the milk, characterizing a nutraceutical effect of supplementation, as it produced milk enriched in iron, an essential mineral in human diets, especially for children who often require supplementation in the first years of life.

Fructosamine is a compound formed by the non-enzymatic binding of glucose to plasma proteins, primarily albumin. It reflects average blood glucose concentrations over approximately two to three weeks, corresponding to the half-life of bovine albumin. Therefore, it is considered a useful marker for assessing glycemic control and energy status in dairy cows [35]. In the present study, a significant effect of treatment and the treatment x day interaction on fructosamine levels was observed, with reduced values in the group supplemented with organic iron on days 16 and 29 compared to the control group. In contrast, the study by researchers [36] found that cows with higher fructosamine levels had higher dry matter intake, better energy balance, and less liver inflammation. However, in the present experiment, the reduction in fructosamine occurred without significant changes in dry matter intake, body condition, or average production indicators. This may be related to the fact that the cows were in the final third of lactation, rather than the transition period, when energy metabolism fluctuates more. Furthermore, Rahko et al. [37] had already observed that cows that experienced significant weight loss in the three weeks prior to sampling showed lower fructosamine levels, indicating that reductions in this variable may be associated with a negative energy balance. However, no weight loss or worsening of production performance was observed at the time of these changes, but in the last week of the study, there was lower milk production in the iron-supplemented cows. The decrease in fructosamine may be a possible adaptive response to iron supplementation, which may have contributed to greater peripheral glucose uptake, reduced circulating blood glucose, and, consequently, lower average fructosamine levels.

Serum activity of the liver enzymes ALT and AST is widely used as a biomarker of liver and/or muscle integrity in cattle [38,39]. Under physiological conditions, their levels tend to remain stable; however, they increase significantly in response to cell injury, inflammation, or metabolic disorders [40]. In the present study, a significant effect of the treatment x day interaction was observed on the levels of these enzymes, with more constant AST values in the control group and a significant increase in AST in the iron group on days 29 and 42. ALT, on the other hand, showed significantly higher values in the iron group, especially on days 29 and 42. AST is an enzyme present in various tissues, such as liver, skeletal muscle, and heart, and its increase may reflect liver or muscle cell damage [40]. On the other hand, ALT is more liver-specific, and therefore, changes in its levels generally indicate direct liver alterations [41]. These findings suggest that, although organic iron promoted an increase in serum iron concentration, its supplementation may have resulted in a transient metabolic overload in the liver, reflected in elevated liver enzyme levels or even hepatocyte damage. According to [41], elevated ALT activity is associated with hepatocellular disorders, since this enzyme is highly liver-specific. Additionally, researchers [40] emphasize that elevations in AST may indicate systemic oxidative stress, which may also have contributed to the results, especially considering the physiological stage of the cows (final third of lactation), a period characterized by high metabolic demand. Therefore, these results reinforce that supplementation of this dose of organic iron reflects detrimental effects on animal health.

Regarding hematological variables, organic iron supplementation had an impact on granulocytes and lymphocytes. Although total leukocytes did not show a significant difference between treatments, lymphocytes on day 42 were lower in cows in the supplemented group, which may be related to mild immunosuppression associated with iron supplementation. According to [42], iron plays a crucial role in T-cell activation through the transferrin receptor (CD71). However, in situations of excess, intracellular iron can trigger the production of ROS, promoting oxidative stress and cell death by ferroptosis, especially in regulatory and CD4+ T cells. This reduces lymphocyte proliferation and activation, as observed in the present study. Additionally, Wang et al. [43] explain that iron exerts ambiguous effects on the immune system: it is essential for the proliferation of immune cells, but excess iron can lead to chronic activation of innate immunity and suppression of the adaptive response, primarily through increased hepcidin levels induced by inflammatory cytokines such as IL-6. This increase in hepcidin reduces iron export by phagocytic cells, sequestering iron in tissues and limiting its functional availability, which may explain the lymphocyte decline observed even with high circulating iron levels.

The increase in granulocyte counts in the iron-supplemented group on day 42, when the experiment was terminated, was noteworthy. Granulocytes, primarily neutrophils, are cells that act as the first line of defense against infection, especially of bacterial origin. The significant elevation of these cells suggests the presence of an inflammatory or infectious stimulus, which may have been exacerbated by iron supplementation. Iron is an essential micronutrient for neutrophil activity, being necessary for processes such as ROS production, NET (neutrophil extracellular trap) formation, and enzyme activation—critical mechanisms for pathogen destruction [42]. Supplementation with organic iron may have altered this balance, making iron available not only to host cells but also potentially to opportunistic pathogens, as mentioned above. This favors the onset of subclinical infections, which, in turn, stimulate granulocyte expansion, as observed in this experiment. A study by researchers [44] investigated the effects of iron and copper supplementation in pregnant cows, and the results showed that iron addition significantly reduced neutrophil phagocytosis capacity, suggesting functional immunosuppression. This dysfunction may lead to a compensatory response by the body, increasing granulocyte production to combat potential subclinical infections. Another study [45], which examined the effects of parenteral iron and copper administration in neonatal calves, found a significant increase in neutrophil counts upon iron utilization, suggesting an activated innate immune response, possibly due to subclinical infections facilitated by increased iron availability for pathogens.

Organic iron supplementation resulted in a significant increase in ROS and TBARS levels combined with elevated SOD activity, indicating an intensification of oxidative reactions, which can lead to exacerbated oxidative stress. These findings are consistent with the review by Wysocka et al. [46], who emphasize that, although iron is essential for processes such as hematopoiesis, energy metabolism, and immune function, excess iron can participate in Fenton-type reactions, promoting the generation of highly reactive hydroxyl radicals and contributing to oxidative tissue damage. The study also highlights that, even in adult cows, an imbalance between bioavailable iron and antioxidant capacity can expose animals to oxidative stress. Similarly, the study conducted by Santos et al. [47] evaluated goats with anemia and respiratory disease treated with parenteral iron, where sick goats showed a significant increase in TBARS levels within 48 h of iron application, which was interpreted as indicative of oxidative toxicity aggravated by the excess availability of free iron. Although the experimental models differ (sick and anemic goats versus healthy lactating cows), both studies point to the potential pro-oxidant effect of iron when supplied in excess or in critical physiological situations, such as infectious diseases or phases of high metabolic demand. Myeloperoxidase (MPO) activity did not show significant differences between the group supplemented with organic iron and the control group, although this was expected, given that iron supplementation had an effect on granulocyte counts. MPO is an enzyme produced by neutrophils during the activation of innate immunity and is involved in the formation of reactive nitrogen and oxygen species. Its stability may indicate the absence of marked systemic inflammation or insufficient stimulation of neutrophil degranulation [48]. Furthermore, PSH levels remained constant, corroborating the findings of researchers [49], who investigated thiol/disulfide homeostasis as a novel indicator of oxidative stress in dairy cows with subclinical endometritis. The same authors observed that, even in the presence of oxidative stress, thiol levels can remain stable due to the body’s intrinsic antioxidant capacity and the action of compensatory systems. Considering that the cows were in the final third of lactation, a less stressful physiological phase from an immunometabolic perspective, it is understandable that the antioxidant system is less challenged to the point of significantly altering these markers [50], which may explain the slight effect on SOD activity.

The combined effects of iron supplementation on immunity, energy metabolism, and oxidative stress help explain its impact from a production perspective. Daily milk production was 6% lower in iron-supplemented cows. The negative effect on milk production was evident in the final phase of the experiment, directly related to cases of mastitis and intestinal peristalsis paralysis. However, according to [8], in which the researchers evaluated supplementation with 30 mg/kg of organic iron (Availa-Fe) during the final third of gestation and early lactation, there was no significant improvement in milk production in cows fed basal diets already rich in iron (282–336 mg/kg dry matter), especially from forages. The authors suggest that, in situations where dietary iron is already sufficient, additional supplementation may be unnecessary and even harmful, since excess iron can induce oxidative stress and harm the cows’ metabolic health, confirming our findings. In our research, using an organic iron source that tends to increase the mineral’s bioavailability ultimately harmed the cows’ health for the reasons already mentioned. This inflammatory condition and the resulting activation of the innate immune system increase basal energy intake, redirecting nutrients and energy that would normally be allocated to milk production to the maintenance of physiological processes related to the immune response and homeostasis [51].

Consistent with our results, researchers [7] conducted a case study to evaluate the effect of an amino acid iron complex (Availa-Fe) on the performance of lactating dairy cows on a commercial farm. The study involved 506 lactating Holstein cows, which were divided into groups receiving either a basal diet (control) or the same diet supplemented with 600 mg/day of iron as an amino acid complex. The results indicated that iron supplementation did not significantly affect milk production, nor did it influence milk composition, corroborating the results found in our study. Therefore, we believe that the observed reduction in milk production in the supplemented group is multifactorial and may be attributed to iron overload, associated oxidative stress, activation of the innate immune system, immunosuppression, and possible energy diversion, compromising the cows’ productive potential; as well as the direct effect of cases of mastitis that lead to reduced milk production in the affected mammary quarter. Therefore, based on our results, organic iron supplementation at a dose of 600 mg/animal/day is contraindicated under these experimental conditions.

The gut microbiota is recognized as one of the main modulators of animal health, actively participating in nutrient digestion, immune regulation, and protection against pathogenic microorganisms [52]. Considering this central role in ruminant health, we investigated how organic iron supplementation could influence the fecal bacterial community of the cows evaluated. Changes in the bovine microbiota have already been reported as a consequence of environmental and nutritional factors [53], and in the case of iron, its availability is crucial for both the host and intestinal bacteria, potentially favoring opportunistic species or reducing important commensal groups [54,55]. In the present study, organic iron supplementation modulated the relative abundance of several bacterial genera. The growth of *Pseudomonas* in iron-rich environments is in line with studies demonstrating its ability to capture iron by siderophores [55]. Such opportunistic expansion can disrupt the balance of commensal anaerobes, favoring pro-oxidative and pro-inflammatory environments within the gut [56]. On the other hand, the loss of *Acinetobacter and Comamonas*, genera associated with microbiota degradation and stability, suggests compromised intestinal ecological resilience [57]. Species-level analysis revealed significant reductions in *Acinetobacter idrijaensis* (−51.6%) and *Comamonas kerstersii* (−53.8%) in supplemented animals, confirming that the loss of relevant commensal groups was consistent. Their reduction in the treatment group suggests that iron supplementation may have selectively suppressed metabolically beneficial taxa, potentially altering fermentation dynamics and nutrient turnover in the distal gut [58].

Cows with dysbiosis exhibited more marked changes, including the disappearance of *Psychrobacter* and an increase in transient colonists (e.g., *Flavobacterium*, *Sporosarcina*) and opportunistic pathogens (e.g., *Brevundimonas*), indicating microbial reorganization in response to iron-induced metabolic stress. *Arthrobacter* species, which are soil-derived *Actinobacteria*, are commonly detected in the bovine gut during feed transition or epithelial turnover events; their relative enrichment might reflect impaired mucosal barrier integrity or altered feed utilization following iron supplementation [59]. The stressful, pro-inflammatory gut environment induced by iron overload can selectively inhibit the growth of obligate anaerobic commensals. This can create an ecological vacuum that permits the detection and/or limited growth of environmental bacteria.

Microbial diversity was notably influenced by the treatment, as beta diversity analysis revealed distinct clustering of cows with dysbiosis, whose alpha diversity was significantly greater than that observed in the control and mastitis groups. This finding contrasts with the classical view that greater diversity indicates better gut health. In dysbiosis situations, this increase may reflect the expansion of opportunistic and potentially pathogenic species to the detriment of stable communities [60], which reinforces the interpretation of ecological imbalance induced by supplementation.

The LDA score confirmed distinct shifts in the fecal microbial composition between experimental groups. In the control group, higher relative abundances of *Olsenella*, *Heyndrickxia*, *Anaeroplasma*, and *Kocuria* were observed, genera associated with carbohydrate fermentation, lactate utilization, and the production of beneficial metabolites such as short-chain fatty acids [52]. Conversely, cows supplemented with organic iron exhibited an increased relative abundance of *Nitratireductor*, a genus involved in nitrogen and nitrate reduction pathways. Members of this genus are capable of producing reactive nitrogen intermediates that can contribute to local oxidative stress and intestinal inflammation [61]. This microbial shift may reflect an adaptive response to altered iron availability, as excess iron promotes oxidative reactions and supports the growth of microorganisms with enhanced detoxification or denitrification capacities [31].

## 5. Conclusions

Chelated iron supplementation in cows in the final third of lactation increased serum and milk levels of the mineral, demonstrating its high bioavailability and nutraceutical potential for milk enrichment. However, the strategy also caused adverse effects, such as immunological alterations consistent with activation of the innate response and possible adaptive immunosuppression, increased markers of oxidative stress, elevated liver enzymes, reduced milk production, and a higher incidence of clinical disorders. Furthermore, supplemental iron intake modulated the gut microbiota, increasing opportunistic and potentially pathogenic microorganisms. Thus, despite the potential benefit to the consumer, supplementation with 30 mg of iron/kg of dry matter (600 mg/animal/day) compromised cow health and performance and is not recommended, as the health and productivity risks outweighed the potential benefits.

## Figures and Tables

**Table 1:** Proximate composition of feedstuffs and micromineral levels in the concentrates.

Variables, %	Silage	Hay	Concentrate	Pelleted Concentrate
Dry matter (%)	27.11	84.75	88.38	87.83
Ash, % DM	4.39	6.86	7.10	5.56
Crude protein, % DM	4.31	2.80	18.50	20.63
Ether extract, % DM	2.97	0.91	2.67	3.80
NDF, % DM	63.86	75.70	23.54	23.37
ADF, % DM	34.10	34.46	10.88	9.60
Minerals in concentrate, ppm	Control	Iron		
Iron	369.86	427.16		
Aluminum	396.94	400.38		
Boron	11.64	14.36		
Copper	24.37	29.05		
Manganese	144.85	166.17		
Zinc	149.79	146.46		

Note: The proportions of the total diet (TMR) are as follows: Corn silage (48.9%), Hay (5.7%), Basal Concentrate (35.94%), and Pelleted Concentrate (9.46%), as detailed in Table S1. DM = dry matter; NDF = neutral detergent fiber; ADF = acid detergent fiber; Ash = mineral matter.

**Table 2:** Hematological and serum biochemical parameters in Jersey cows supplemented with organic iron.

Variable	Control	Iron	SEM	P: Treat	P: Treat × Day
***Hematology***					
Leukocytes (×10^3^/µL)	7.19	7.9	0.65	0.67	0.51
Lymphocytes (×10^3^/µL)				0.12	0.05
d1	4.13	4.18	0.21		
d16	4.76	4.65	0.24		
d29	4.42	3.6	0.19		
d42	4.42 ^a^	3.28 ^b^	0.19		
Mean	4.54	3.84	0.18		
Granulocytes (×10^3^/µL)				0.05	0.01
d1	1.62	1.73	0.09		
d16	2.46	2.82	0.15		
d29	1.9	2.34	0.12		
d42	2.04 ^b^	3.73 ^a^	0.14		
Mean	2.13 ^b^	2.96 ^a^	0.12		
Monocytes (×10^3^/µL)	0.83	1.09	0.24	0.52	0.75
Erythrocytes (×10^6^/µL)	5.23	5.39	0.07	0.96	0.97
Hemoglobin (g/dL)	9.49	9.45	0.11	0.97	0.97
Hematocrit (%)	26.7	26.7	0.84	0.95	0.93
Platelets (×10^3^/µL)	300	342	12.6	0.22	0.15
***Serum biochemistry***					
Albumin (g/dL)	3.43	3.18	0.15	0.32	0.21
Creatine kinase (U/L)	277	278	3.78	0.92	0.89
Cholesterol (mg/dL)	159	148	2.89	0.41	0.33
Cholinesterase (U/L)	175	179	6.42	0.91	0.94
Creatinine (mg/dL)	0.61	0.56	0.03	0.83	0.76
Ferritin (µg/dL)	478	470	3.65	0.81	0.64
Iron (µg/dL)	187	233	5.41	0.01	0.01
Fructosamine (µmol/L)	270	243	4.22	0.01	0.01
GGT (U/L)	34.1	35.3	2.02	0.91	0.96
C-reactive protein (mg/dL)	13.1	12.8	0.21	0.97	0.93
UIBC (µg/dL)	173	189	4.05	0.05	0.01
Total protein (g/dL)	6.70	6.91	0.21	0.89	0.87
AST (U/L)	97.7	105	3.62	0.35	0.05
ALT (U/L)	27.2	32.1	1.97	0.09	0.03
Urea (mg/dL)	33.7	30.2	2.45	0.78	0.86
Globulin (g/dL)	3.27	3.43	0.18	0.65	0.72

Note: Different letters within the same row indicate statistical differences between groups (*p* ≤ 0.05) or trends (0.05 < *p* ≤ 0.10). GGT = gamma-glutamyl transferase; UIBC = unsaturated iron-binding capacity; AST = aspartate aminotransferase; ALT = alanine aminotransferase. Treat = treatment.

**Table 3:** Oxidative status in the blood of Jersey cows supplemented with organic iron.

Variable	Control	Iron	SEM	P: Treat	P: Treat × Day
ROS (% fluorescence intensity)				0.05	0.023
d1	10.7	13.9	0.59		
d16	9.45	10.8	0.38		
d29	8.88 ^b^	13.2 ^a^	0.32		
d42	8.09 ^b^	13.9 ^a^	0.29		
Mean	8.87 ^b^	12.4 ^a^	0.35		
TBARS (nmol/mL)				0.03	0.001
d1	9.04	9.09	0.08		
d16	10.9 ^b^	14.8 ^a^	0.48		
d29	13.1 ^b^	31.9 ^a^	2.14		
d42	13.2 ^b^	30.4 ^a^	2.01		
Mean	12.4 ^b^	25.4 ^a^	1.97		
MPO (µM)	2.38	2.49	0.16	0.86	0.75
PSH (µmol/L)	3.67	3.74	0.09	0.81	0.57
SOD (U/mg protein)				0.46	0.05
d1	0.30	0.31	0.02		
d16	0.25	0.25	0.01		
d29	0.26	0.24	0.02		
d42	0.24 ^b^	0.30 ^a^	0.01		
Mean	0.25	0.27	0.01		

Note: Different letters within the same row indicate statistical differences between groups (*p* ≤ 0.05) or trends (0.05 < *p* ≤ 0.10). ROS = reactive oxygen species; TBARS = thiobarbituric acid reactive substances; MPO = myeloperoxidase; PSH = total thiols; SOD = superoxide dismutase. Treat = treatment.

**Table 4:** Effects of organic iron supplementation on milk yield and composition, dry matter intake, and feed efficiency of cows in late lactation.

Variable	Control	Iron	SEM	P: Treat	P: Treat × day
Milk yield, kg	25.1	23.6	0.69	0.52	0.01
4% FCM	26.6	25.3	0.62	0.71	0.07
Dry matter intake, kg	16.2	17.8	0.81	0.89	0.56
Feed efficiency, kg/kg	1.54	1.34	0.15	0.35	0.48
Fat, %	4.41	4.48	0.23	0.94	0.90
Protein, %	3.72	3.65	0.21	0.85	0.89
Lactose, %	4.63	4.64	0.03	0.98	0.95
Total solids, %	12.76	12.77	0.28	0.97	0.94
Urea, mg/dL	15.1	14	0.74	0.72	0.61
SCC (×10^3^/mL)	130	272	16.7	0.68	0.05
d1	121	98.4	12.8		
d16	127	105	14.1		
d29	137	205	19.4		
d42	132 ^b^	508 ^a^	28.6		
Milk iron (µg/dL)	10.7 ^b^	12.8 ^a^	0.71	0.05	0.27

Note: Different letters within the same row indicate statistical differences between groups (*p* ≤ 0.05) or trends (0.05 < *p* ≤ 0.10). SCC = somatic cell count; 4% FCM = 4% fat-corrected milk. Treat = treatment.

## Data Availability

The data are with the authors and may be made available upon request.

## Supplementary Materials

The following supporting information can be downloaded at: https://www.mdpi.com/article/10.3390/ani15233373/s1, Table S1: Proximate composition and mineral levels in the experimental diet; Figure S1: Relative abundance based on the Greengenes database showing the 20 most abundant microbial species in the feces of cows supplemented with iron compared with the control group.
