# Supplementary material for: Organic Iron Supplementation in Cows and Its Impacts on Animal Health and Production
Source: Animals (Basel). 2025 Nov 21;15(23):3373. doi: 10.3390/ani15233373 (PMC12691437; doi:10.3390/ani15233373)
Supplement: Supplementary file 1 [file animals-15-03373-s001.zip › animals-3964198-supplementary.pdf]

Table S1. Proximate composition and mineral levels in the experimental diet.

Composition of the diet supplied in individual feeders (kg of DM/day)		
Ingredient	***Intake (kg DM/day)***	***% of total DM***
Corn silage	*9.12*	*48.93*
Tifton 85 hay	*1.06*	*5.67*
Ground corn (fine)	*2.78*	*14.89*
Soybean meal	*2.34*	*12.56*
Wheat bran	*0.39*	*2.08*
Soybean bran	*0.75*	*4.02*
Sodium bicarbonate	*0.13*	*0.69*
Urea	*0.05*	*0.28*
Premix ALFA[1]	*0.03*	*0.17*
Limestone	*0.11*	*0.57*
Dicalcium phosphate	*0.05*	*0.28*
Sodium chloride	*0.08*	*0.40*
ALFA 18% ROBO concentrate	*1.76*	*9.46*
Calculated levels of macrominerals in the total diet		
Mineral	***Absorbable supply (g/day)***	***Concentration in the diet (%)***
Calcium (Ca)	74.0	0.61
Phosphorus (P)	49.4	0.38
Magnesium (Mg)	7.1	0.23
Chlorine (Cl)	75.1	0.45
Potassium (K)	162.3	0.97
Sodium (Na)	64.4	0.38
Sulfur (S)	36.7	0.20
Calculated levels of microminerals in the total diet		
**Mineral**	***Absorbable supply (mg/day)***	***Concentration in the diet (ppm)***
Iron (Fe)	50.720	27.21
Copper (Cu)	10.920	14.65
Zinc (Zn)	59.880	57.95
Manganese (Mn)	3.960	59.95
Selenium (Se)	5.690	0.31

[1]Guaranteed levels: Monensin 7,500.00 mg/kg; Sulfur (min) 100.00 g/kg; Magnesium (min) 200.00 g/kg; Cobalt (min) 300.00 mg/kg; Copper (min) 5,600.00 mg/kg; Iodine (min) 310.00 mg/kg; Manganese (min) 20.00 g/kg; Selenium (min) 160.00 mg/kg; Vitamin A (min) 2,500,000.00 IU/kg; Vitamin D_3 (min) 750,000.00 IU/kg; Vitamin E (min) 12,500.00 IU/kg; Zinc (min) 23.75 g/kg.

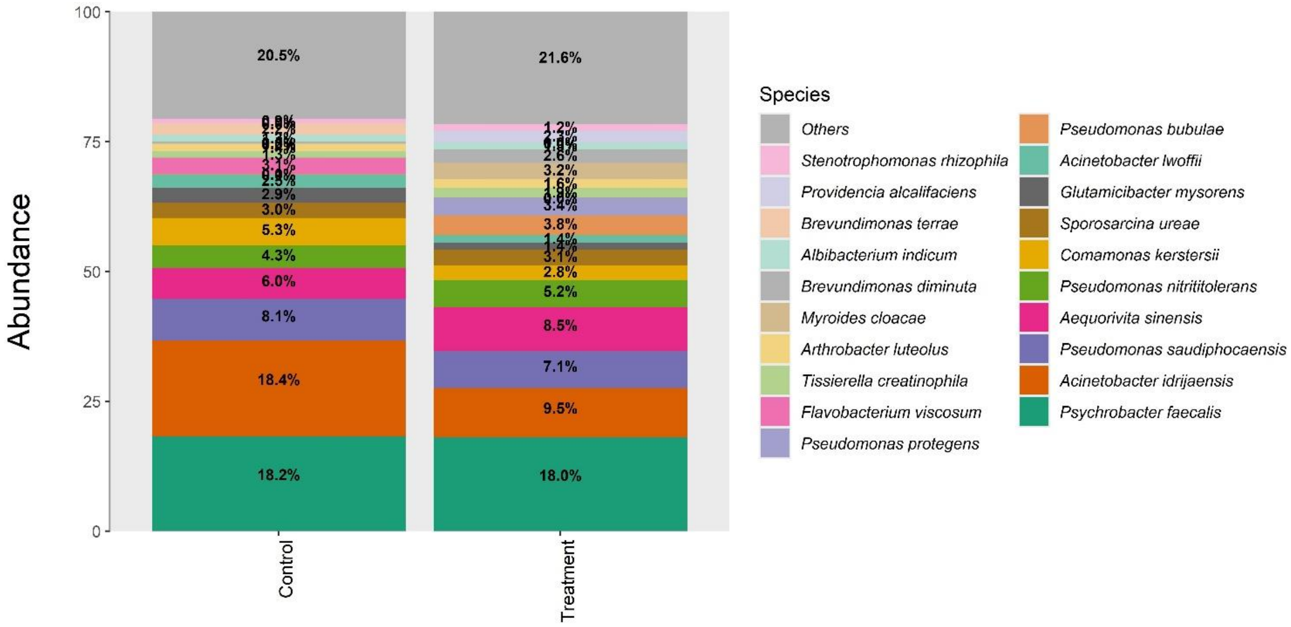

Figure S1. Relative abundance based on the Greengenes database showing the 20 most abundant microbial species in the feces of cows supplemented with iron compared with the control group.
